# 3D Printed Hollow Off-Axis Profiles Based on Carbon Fiber-Reinforced Polymers: Mechanical Testing and Finite Element Method Analysis

**DOI:** 10.3390/polym13172949

**Published:** 2021-08-31

**Authors:** Martina Kalova, Sona Rusnakova, David Krzikalla, Jakub Mesicek, Radek Tomasek, Adela Podeprelova, Jiri Rosicky, Marek Pagac

**Affiliations:** 1 Center for Advanced Innovation Technologies, VSB-TU Ostrava, 17. Listopadu 2172/15, 708 00 Ostrava-Poruba, Czech Republic; radek.tomasek@vsb.cz (R.T.); adela.podeprelova@vsb.cz (A.P.); 2 Department of Production Engineering, Faculty of Technology, Tomas Bata University in Zlin, Vavreckova 275, 760 01 Zlin, Czech Republic; rusnakova@utb.cz; 3 Center of 3D Printing Protolab, Department of Machining, Assembly and Engineering Technology, Faculty of Mechanical Engineering, VSB-TU Ostrava, 17. Listopadu 2172/15, 708 00 Ostrava-Poruba, Czech Republic; david.krzikalla@vsb.cz (D.K.); jakub.mesicek@vsb.cz (J.M.); 4 Orthopedic Prosthetics Frydek-Mistek, Dr. Janskeho 3238, 738 01 Frydek-Mistek, Czech Republic; jiri.rosicky@inventmedical.cz

**Keywords:** composite polymer materials, carbon fibers, hollow profile, 3D printing, fused deposition modeling, FEM analysis, SEM analysis

## Abstract

The aim of the paper is to design, manufacture, and test an off-axis composite profile of circular cross-section. Composite profile based on continuous carbon fibers reinforcing the onyx matrix, i.e., a matrix that consists of nylon and micro carbon fibers, was produced by fused deposition modeling (FDM) method. A buckling test of the six printed composite specimens was performed on a tensile test machine. The values of the experiment were compared with the values of the computational simulation using the Finite Element Method (FEM) analysis. The mean value of the experimentally determined critical force at which the composite profile failed was 3102 N, while the value of the critical force by FEM analysis was calculated to be 2879 N. Thus, reliability of the simulation to determine the critical force differed from the experimental procedure by only 7%. FEM analysis revealed that the primary failure of 3D printed composite parts was not due to loss of stability, but due to material failure. With great accuracy, the results of the comparison show that it is possible to predict the mechanical properties of 3D printed composite laminates on the basis of a theoretical model.

## 1. Introduction

Due to their excellent mechanical properties, such as high specific stiffness and strength, fiber-reinforced polymer composites (FRP) are used in structural applications, mainly in the automotive industry (luxury bodies, intake manifolds, interior and safety elements, and axles), aerospace industry (refractory parts, internal elements, and rotor blades), or for the things in everyday life (skis, tennis rackets, safety helmets, musical instruments, or outdoor items) [1,2,3]. In the field of medicine, FRPs appear mainly as a structural part of rehabilitation aids, where they replace traditional materials such as dural. The use of composite materials meets the requirements for lightening, increasing the stability of the structural element, load-bearing capacity, and design [4]. A major disadvantage of conventional carbon fiber-reinforced composite (CFRP) production methods is the high acquisition costs, such as pultrusion lines or a winding machine. It is more suitable for large-series production and products with a constant cross-section [5,6]. Therefore, there is a growing interest in new processes based on additive production. 3D printing enables the production of complex geometries, faster production times, and the production of accurate and reproducible parts without the need to produce expensive molds [7,8]. The prints are light, durable, geometrically accurate, and, thanks to minimal material waste, 3D printing technology is also environmentally friendly [9].

Fused Deposition Modeling (FDM), one of methods of 3D printing, is based on heating and subsequent melting of a thermoplastic fiber to a temperature higher than the glass transition temperature. This is followed by controlled extrusion of the plastic through a heated nozzle onto the printing surface [10,11]. During the printing process, no pressure is exerted during the laying of the polymer layer. This creates unfilled gaps, pores, or cavities in the material. The high pore content results in much lower stiffness and strength of the material compared to traditionally produced FRP composites. When printing a curved part, there is also a risk of twisting or breaking the fiber bundle [9,12]. However, in addition to temperature and material selection, the productivity of the 3D printing process and the quality of the final parts depend on other factors, including the geometric complexity of the part, fill density, layer thickness, print speed, or fiber orientation [13,14,15]. As with hand-lay-up composites, there is a strong dependence of the properties of the laminate material on the orientation of both short and continuous fibers. The strength and stiffness of the laminate are maximized when the fiber orientation is parallel to the direction of loading [16,17]. Due to the limited mechanical properties of polymer-based 3D prints, FDM-printed parts are only used as prototypes, not as functional components. The mechanical properties of polymer prints can be improved by adding high-strength fibers (short, continuous) to the polymer matrix during the FDM process. The design of composite printed parts is thus freer in contrast to conventional techniques for the production of composite materials [18,19,20].

There are a number of FDM 3D printers on the market that are capable of producing composite parts. For example, Markforged, USA, has developed the Mark One/Two printer, which can produce composites with continuous fiber reinforcement. The Mark Two printer is supported by Eiger’s own specialized software [21]. This printer uses two extruders and two printheads to separately extrude the die and fibers in the desired positions. The design of the 3D printer allows the placement of continuous fiber reinforcement as needed through the layer-by-layer application process [9,21]. Another commercially available printer is the Markforged X7, which is capable of printing continuous fibers only on the inside of printed parts, and the continuous fiber material contains additives that facilitate the FDM printing process. This causes a reduction in longitudinal and flexural strength compared to raw carbon fibers [22,23].

Increasingly, scientists are evaluating the mechanical properties (tensile and compressive, bending, impact, fatigue, or creep) of 3D printed composites. The tensile and flexural properties of 3D printed fiber-reinforced polymer composites have been discussed, for example, by Justo et al. [24], Korkees et al. [25], Mohammadizadeh et al. [26], and Pertuz et al. [27]. In many cases, such as in the article by Yasa et al. [28], it has been found that adding reinforcement to the thermoplastic matrix increases the tensile strength, but only to a certain extent. The problem with the FDM method is that pores are formed in the structure, which degrade the mechanical properties. The mechanical properties of CFRP based on onyx matrix, the influence of fiber orientation and of defects on the properties of printed structures by Wickramasinghe et al. [29] were studied. Although the tensile strength of the composite (CF/nylon) was increased by the fibers, the addition of additional layers of fibers increased the cavity content, causing poor tensile modulus. The addition of continuous fibers to the thermoplastic increased the tensile and flexural strength, but the compressive strength was reduced, again due to defects caused by printing.

Components in industries often contain structural holes. Holes are places of stress concentration which affect the strength and reliability of the product. Pollen et al. [30] and Sanei et al. [31] investigated the effect of stress concentration on 3D printed parts using tensile samples around the open hole area. Prajapati et al. [32] investigated the effect of HSHT continuous glass fiber reinforcement on the open hole tensile strength of 3D printed parts. Onyx was used as a polymer matrix material. It has been found that the fiber reinforcement in the onyx matrix increases the open hole tensile strength, but it also increases the specimen weight and printing time of the final part. Ekoi et al. [33] compared the mechanical properties (tensile, flexural, and fatigue) of woven continuous carbon fiber composites printed using FDM and nonwoven printed composites (unidirectional and multidirectional fibers), along with woven composites, and also composites reinforced with chopped carbon fibers (onyx). The maximum tensile strength (714 MPa) achieved for composites was highest for unidirectional nonwoven composites. The lowest tensile value was achieved at the multidirectional nonwoven composite (248 MPa). The maximum flexural strength of unidirectional (nonwoven) composites was 407 MPa, and for woven composites, the flexural strength was 251 MPa. Woven carbon fiber composites achieved the best fatigue strength. Therefore, these materials have great potential for more demanding applications (medical and sports devices). Saghir et al. [34] investigated the effects of constituent materials (particle reinforcement, cut glass, glass fiber, and resin) on the axial tensile and the hoop tensile strength of particulate FRP composite pipes. Three specimens for each type of reinforcement and two types of tests (axial tensile test and hoop tensile test) were selected. Saghir showed that the inclusion of a higher proportion of particulate fillers/reinforcements, such as sand or other components, can reduce production costs, but also causes a reduction in both axial and hoop strength. Research has shown that the ratios of material components have a measurable impact on pipe performance.

An interesting conclusion was reached by the research of Saharudin et al. [35]. Both FDM and CFF (Continuous Filament Fabrication) 3D printing technologies were compared, and it was shown that the addition of carbon fibers alone is not a factor determining the high mechanical properties, the method of fiber supply depending on the 3D printing method is also important.

Latest studies on the mechanical properties of 3D composites with printed continuous fiber have been summarized by Zhuo et al. [36]. The addition of continuous fiber reinforcement improves mechanical properties, but at the cost of increased complexity and cost. The fiber volume fraction must be high enough and the defect content low enough for printed products to be used in real engineering structures. To improve the mechanical properties of printed composites, it is necessary to understand the relationships between the AM (Additive Manufacturing) process, the structure of printed parts and their mechanical performance [37].

From the research to date, it can be stated that the problem with composites is to determine their static and dynamic mechanical properties in advance. To obtain more detailed information on mechanical values, composite materials must be tested during their production, or even on the finished product. This is, of course, time consuming and expensive. This manufacturing process can be facilitated by unique software for advanced modeling and simulation of the structures and properties of a given material. Currently, only a small percentage of people are involved in modeling composite structures using software. However, a number of publications are available on the numerical prediction of the mechanical properties of different types of composite materials [38]. Gao et al. dealt with the numerical prediction of mechanical properties of rubber composites reinforced with short aramid fibers at large deformation. Samples with two different fiber lengths and three different fiber volume fractions were subjected to mechanical testing. To predict the mechanical response of the rubber composite, Gao proposed a computational model based on the finite element method. The results obtained by experiment and by numerical simulation in Digimat-FE software (Finite Element—describing the behavior of the material from a microview based on FEM) were compared to verify the reliability of the FE-model. The results were almost identical. It was thus concluded that, with this method, it is possible to obtain a model with randomly dispersed fibers with a high-volume fraction of fibers, and that with the numerical method, it is possible to obtain the mechanical properties of a rubber composite under high deformation [39]. Gohari et al. [40] dealt with the analysis of the failure location of internally pressurized laminated ellipsoidal woven composite domes (CFRP). The experimental and numerical results confirmed the analysis that the deformation occurs locally rather than uniform. Potluri et al. [41], for a change, used modeling to predict the mechanical properties of a natural fiber-reinforced composite. He dealt with the prognosis of the value of the Young’s modulus of elasticity in tension and the modulus of elasticity in shear of a given material. He compared the results obtained analytically, numerically, and experimentally. He wanted to determine which model can accurately predict the values of elastic and shear properties of composite materials reinforced with natural fibers. By comparing the analyses, Potluri showed that all models show a very good correlation for the modulus of elasticity in tension and shear. FE models were further implemented in ANSYS software. Furthermore, Elmarakbi et al. [42] studied the modeling in Digimat, which was used in modeling hybrid glass profiles with a polyamide matrix (PA6) reinforced with graphene plates. In his work, he investigated the impact resistance of this material in a hierarchical modeling of a hybrid composite material consisting of short matrix-reinforced glass fibers and graphite plates. The multistep method uses both the medium homogenization method and the finite element FE technique.

The main contribution of our paper lies in the expansion of knowledge about the behavior of composite off-axis profiles of carbon fibers produced by an unconventional method of 3D printing. In many applications, due to low-series production, it is not worth investing in expensive equipment, such as pultrusion lines or winding machines, and therefore the results experimentally obtained and verified by FEM are a valuable source of information.

The computational method helps us to understand the mechanisms of continuous printing in terms of local stress distribution. The absence of literature on the production of hollow off-axis composite profiles was a challenge for this paper. After considering the available variants, we chose a technology suitable for low-series production and also economically available for the production of profiles.

## 2. Materials and Methods

### 2.1. Design and Manufacture of an Off-Axis CFRP Profiles

**Figure 1:**
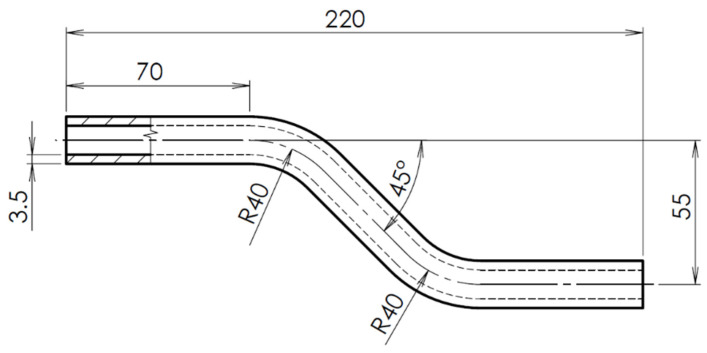
Design of an off-axis hollow CFRP profile.

The geometry of the sample was designed as a hollow off-axis profile of a circular cross-section using the commercial 3D modeling software SolidWorks 2020 (Dassault Systèmes, Vélizy-Villacoublay, France). This shape element is found, for example, in medical supplies and aids (sticks and crutches), as part of the body structure. This shaped element helps to better transfer compressive loads to the base of the aids and dampens vibrations. The dimensions of the composite profile were given both from an economic point of view, as well as the possibilities of printing itself and the minimum printable thicknesses, so that the walls of the hollow circular profile did not collapse. With a sample diameter of 18 mm, a wall thickness of at least 3.5 mm was required. At this wall thickness, a sufficient coating of the reinforcing fibers with an onyx matrix was achieved. Figure 1 presents a schema of the design of composite profile. 

**Figure 2:**
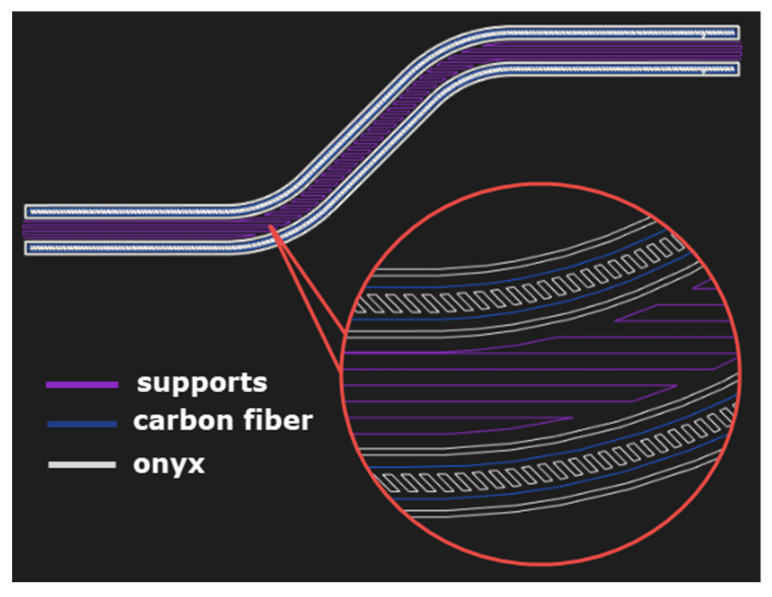
Layout of matrix and reinforcement for 3D printing of CFRP profiles.

The Markforged X7 3D printer (Markforget, Watertown, MA, USA) is designed from the ground up to print composite continuous fiber parts. It contains a reinforced two-nozzle system that supports the printing of the matrix, and at the same time continuous carbon or other reinforcing fibers. The laser scans the part during printing to ensure maximum dimensional accuracy [43]. A tough onyx matrix (basic material) reinforced with carbon fiber was chosen for printing composite prototypes using the FDM/FFF method on a 3D printer. The 2D design with matrix and reinforcement layout was performed in the Eiger software (Figure 2), which is an accessory of the 3D printer.

Onyx is a thermoplastic that is up to 1.4× stronger than ABS. The strength of the onyx can be further increased by reinforcement in the form of continuous fibers. Onyx is chemically and thermally resistant, but must be stored in a dry box for protection against moisture to prevent deterioration [44]. Onyx fibers with a diameter of 1.75 mm and carbon fibers with a diameter of 0.34–0.38 mm were supplied by Markforged. The mechanical properties (from datasheet) of the material used (onyx/CF) for 3D printing are given in Table 1.

The print parameters were also set in the Eiger Markforged software. The print layer height was set to 0.125 mm. The number of layers was then determined to a total of 144. The time required for the preparation and printing of the 6 composite profiles was calculated to be about 63 h. An overview of the printing parameters for one sample is given in Table 2.

### 2.2. Testing of an Off-Axis CFRP Profile

**Figure 3:**
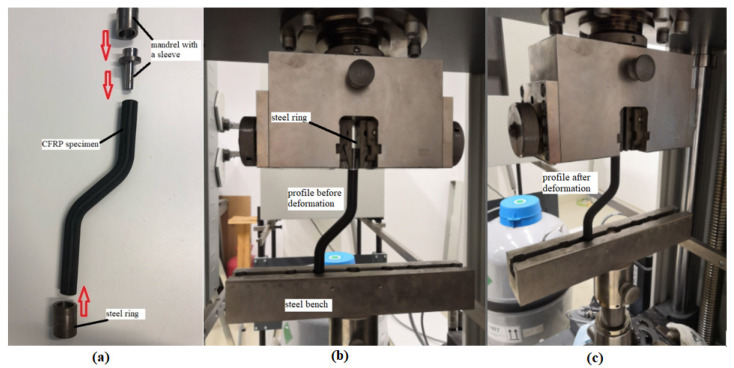
Mechanical testing of an off-axis hollow CFRP profile; (**a**) “tailor-make” a jig to attach the composite profile to the upper and lower jaws grips, (**b**) CF profile before mechanical testing, (**c**) CF profile after mechanical testing.

The composite CFRP profile (six specimens) buckling test was performed on a Zwick/Roell Z150 universal tensile test machine (ZwickRoell, Ulm, Germany) [45]. This testing machine is fully automated and uses a hydraulic control mechanism to transmit the gradual separation of the jaws at a constant speed. Load-cell of the tensile test machine is calibrated regularly every two years. The mechanical properties of this composite materials class have not been comprehensively studied to date. In this case, it is more precisely a modified buckling test as, due to the shape of the test part, both buckling and the bending occur during loading. For testing off-axis prototypes, it was necessary to tailor-make a jig to attach the composite profile to the upper and lower jaws grips of the tensile test machine. At the top of the machine, the specimens were slid onto a steel mandrel with a sleeve. A steel bench was placed in the lower jaw with holes for accommodating composite rods provided with a steel ring against slipping in the bench. The sample was then set up on the tester to ensure adequate alignment. The load cell was zeroed with each new measurement. Tests were carried out at a deformation speed of 5 mm/min. Load was applied to the samples until the maximum failure force could be evaluated. The modification of the test set-up is shown in Figure 3. By comparing Figure 3b (before loading) and Figure 3c (after loading), it can be seen how the specimen bends downwards towards the steel bench after loading.

## 3. Results and Discussion

There are no uniform test conditions for polymer composites due to their variable morphology and chemical composition; they are regulated only in a framework by the standard. Therefore, the specific method of a particular material is governed by professional discretion, taking into account how the future product will be stressed during its use [46].

In some structures, it is possible to find cases where the load-bearing capacity is not critical in terms of material strength, but in terms of stability. The issue of loss of stability, which most often occurs under pressure, bending, torsion, or a combination thereof, deals with the stability of structures. Stability is most often solved for open and closed profiles or for thin-walled beams. One of the basic cases of stability, which is solved most often, is the so-called buckling. The buckling occurs when the slender member is loaded by a compressive axial force. The pressure acts on the reinforced layer in the direction of the fiber axes until it breaks due to the loss of stability of these fibers. The degree of fiber resistance depends on the fiber crimp and the level of interfacial cohesion. With good adhesion of the fiber-matrix interface, fracture occurs due to shear (coordinated deflection of the fibers after exceeding the critical value of the load), and with poor adhesion due to delamination. Under compressive loading, it is difficult to maintain a uniform tension throughout the test specimen throughout the test. Changing the wall thickness of the sample leads to differences in resistance to loss of stability [47,48]. A combination of pressure and bending is used to calculate the critical force of off-axis hollow sections, so-called geometrically imperfect rods. The buckling test is one of the commonly used mechanical tests, which are based on the deformation of a test specimen by pressure to determine the critical force of stability failure. The test specimen is clamped in the jaws of the tensile testing machine, where it is loaded with a constant force, usually until failure (collapse of the structure) [48].

### 3.1. Buckling Test of Hollow Composite Profiles

#### 3.1.1. Graphic Evaluation of the Buckling Test of Hollow Composite Profiles

**Figure 4:**
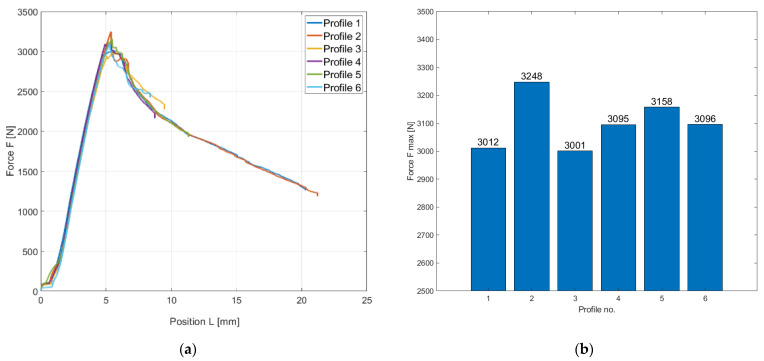
Graphical evaluation buckling test of CFRP profiles produced using 3D printing; (**a**) a line graph of the dependence of the maximum force on the deformation shift, (**b**) a bar graph of the achieved maximum forces for individual profiles.

The result of the buckling test was a stress–strain diagram of the material, which is a curve of the dependence of the load force on the profile displacement. The data were plotted in MATLAB R2019b. Two types of graphs were used for graphical evaluation in Figure 4. A line graph (Figure 4a) showing the dependence of load force on the displacement (compression) of the test profile. The second bar graph (Figure 4b) shows the values of maximum failure strength for each profile. As the hollow sections do not have the shape of a straight bar, they have been subjected to both buckling and bending forces. During testing, the profiles were subjected to compressive forces, but graphs are plotted in absolute values.

From both graphs, it is evident that both the course of the test and the resulting values of the maximum forces of the individual composite profiles are very balanced with one another. This is due to the 3D printing technology, which guarantees the accuracy and reproducibility of parts while achieving repeatable values of mechanical properties. The maximum force for the 3D printed composite profiles was approximately in the range of 3000–3250 N at a displacement (deformation by compressive force) of approximately 5 mm.

**Figure 5:**
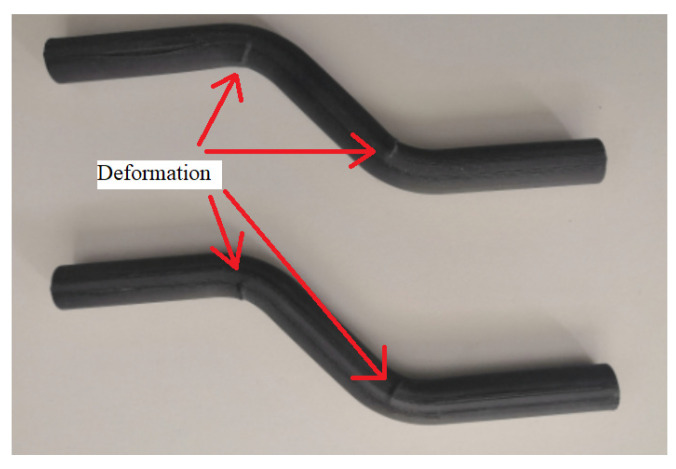
CFRP profiles deformation after buckling test.

During loading, the specimens bent at the area of their curvature. At these points, there was a visible deformation of the structure (surface failure of the material), as indicated in Figure 5. Here, it can be assumed that there was not a failure of stability, but failure of strength. After unloading, the profiles partially returned to their original state as were before the load.

#### 3.1.2. Statistical Evaluation of the Buckling Test of Hollow Composite Profiles

Composite materials show a greater scatter of material characteristics than is the case with conventional materials. Therefore, statistical analysis is an essential part of their evaluation.

Table 3 shows the results of experimental testing of 3D composite profiles on buckling with evaluation of the maximum force (critical force) at which the stability of the samples is disturbed. The table is supplemented by statistical characteristics (arithmetic mean, standard deviation, and coefficient of variation).

### 3.2. Analysis of Composite Profiles Using FEM

FEM analysis of the prototypes printed on a 3D printer were performed in Ansys ADPL 18.2 software. The structure of unidirectionally reinforced composite elements with bends was more complex for modeling and analysis. Software designed specifically for this application is still being developed. Onyx wall was considered as an isotropic material with constants according to the Markforged material sheet [44]. The composite part was considered as a transversely isotropic material. The core elements coordinate system was set to respect fiber direction in real tube thus the material model longitudinal direction respects the fiber direction. An overview of material properties is given in Table 4.

#### Analytical Relationships for Long Fiber Unidirectional Composites

The material model constants were determined by analytical relationships for long fiber unidirectional composites. Here, *μ_f_* is Poisson´s ratio of fiber, *μ_m_* is Poisson´s ratio of matrix, *E_f_* is fiber Young’s modulus, *E_m_* is matrix Young’s modulus and *v_f_* is fiber volumetric content.

- Longitudinal Young’s modulus: 46,522 MPa

The ROM approach (Rule of Mixture) for the calculation assumes an idealized state of the composite—continuous reinforcing fibers of the equal diameter, perfect bonding of the fibers and the matrix or the equal strain of the composite in the longitudinal direction (Equation (1)) [50].

(1) $$E_{L}=v_{f}\cdot E_{f}+v_{m}\cdot E_{m}$$

- Poisson’s ratio: 0.35

The simplicity and accuracy of the ROM was confirmed by several studies comparing the ROM results of analytical and experimental findings (Equation (2)) [51].

(2) $$\mu_{LT}=v_{f}\cdot \mu_{f}+v_{m}\cdot \mu_{m}$$

- Transversal Young’s modulus: 5646 MPa

The relevant Halpin–Tsai model was utilized for the transversal elastic constants of composites. Equations (3) and (4) is a control formula of the H–T model, ksi = 1 [41].

(3) $$E_{T}=\frac{1+\xi \cdot \eta \cdot v_{f}}{1-\eta \cdot v_{f}}\cdot E_{m}$$

(4) $$\eta =\frac{\frac{E_{f}}{E_{m}}-1}{\frac{E_{f}}{E_{m}}+\xi }$$

- Transversal Poisson’s ratio: 0.59

The Clyne model (Equations (5)–(7)) allows a simple calculation. The accuracy of this model is affected by the input variable Et [52].

(5) $$\mu_{TT^{\prime }}=1-\mu_{TL}-\frac{E_{T}}{3\cdot K}$$

(6) $$\mu_{TL}=\mu_{LT}\cdot \frac{E_{T}}{E_{L}}$$

(7) $$K={\left(\frac{v_{f}}{K_{f}}+\frac{v_{m}}{K_{m}}\right)}^{-1}$$

- Shear modulus: 2024 Mpa

Inverse rule of mixture (parallel Reuss model in Equation (8)) gives the lowest estimation [53].

(8) $$G_{LT}={\left(\frac{v_{f}}{K_{f}}+\frac{v_{m}}{K_{m}}\right)}^{-1}$$

**Figure 6:**
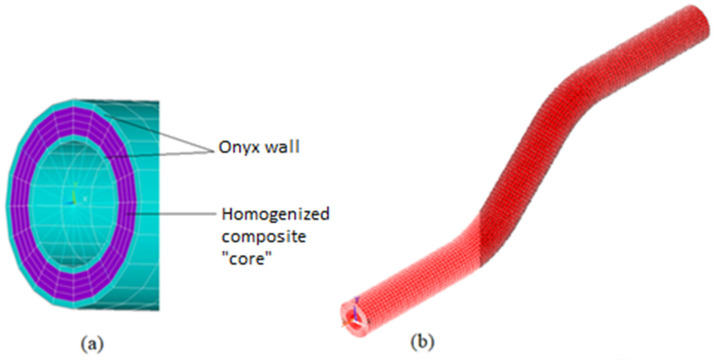
(**a**) The cross-section of the 3D printed prototype model; Onyx walls (blue), composite “core” (purple); (**b**) Final mesh of composite profile.

The component was modeled (Figure 6) as consisting of two parts, an onyx wall and a homogenized composite “core”. The mesh (Figure 6a) shown is for illustration only. The final mesh on which the calculation was performed is shown in Figure 6b.

**Figure 7:**
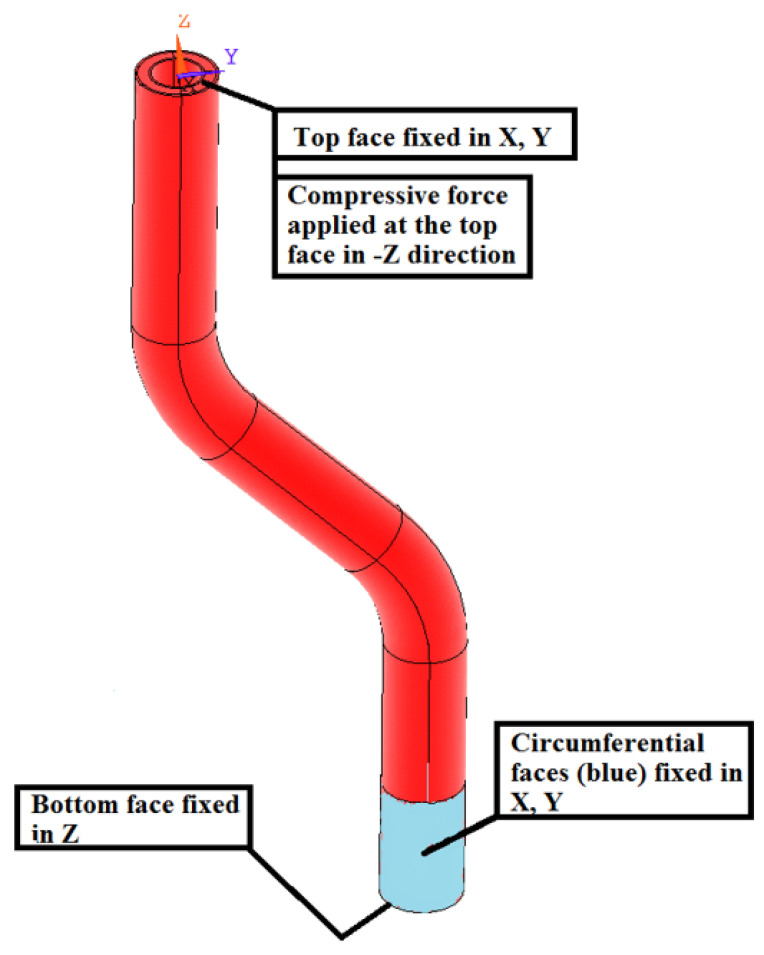
FEM analysis—boundary conditions.

Volumetric linear solid elements (Solid 185 in ANSYS library) were used in the modeling. Some boundary conditions also required the use of Multi-Point Constraint (MPC) elements, specifically MPC 184. The elementary coordinate systems of the composite part were rotated with respect the direction of the fibers. The boundary conditions were set to be as close as possible to the real situation during testing, as well as for the profiles produced by manual methods. In Figure 7, the bottom part of the tube (the portion in the clamps) was fixed in all directions. Then, the top end of the tube was fixed in lateral directions and a compressive force was also applied. 

The sufficient mesh density was obtained and checked by a sensitivity analysis. The final mesh consisted of 28,512 elements and 43,020 nodes. Doubled element count led to marginal (0.1%) change in results, hence the depicted mesh was considered as sufficient.

First, a Linear Buckling (LB) or Linear Bifurcation Analysis (LBA) was performed. In LBA, the structure is considered ideal, without any imperfections and material or geometric nonlinearities. The result are the eigenvalues corresponding to the multipliers of the applied force when the loss of stability is reached, and the shapes of the individual modes of loss of stability (MLS). It is important to realize that MLS only shows the displacement ratios of the structure, and does not represent the real deformation of the structure under loss of stability. The MLS serves only to illustrate the expected deformation of the structure at the edge of stability. From the above, it is clear that LBA overestimates the capacity of structures. In general, a minimum overestimation of 15% is reported. The degree of overestimation, however, depends on the geometry and simplifications of the structure.

The lowest calculated critical force was 18,513 N, which is about 500% higher than the critical force determined from the experiment. Considering the above, it follows that the primary failure of the structure was not achieved due to the loss of stability, but rather the failure of the material. Therefore, a static analysis was subsequently performed.

For evaluating the static analysis, the limit states at which the failure occurs were determined for the walls and the composite part. For the given states, the stress limit values were analytically determined on the basis of the material sheets, and the force required to reach the individual limit states was determined by means of static analyses (see Table 5).

It can be seen from Table 5 that, for states 4 and 5, the limit state (LS) is reached at a lower force than determined experimentally. To achieve LS 1, the force required is 25% greater than that obtained by the experiment. LS 2 and LS 3 are unlikely to occur, as they require relatively more force than the experiment, and other LS will fail much sooner. The critical force was determined from this static analysis.

LS 1, 4, and 5 were considered to be realistically achievable limit states. Subsequently, the average value of force (F_p_) to achieve them was calculated. The value of the average force was 2879 N. Next, the difference F_p_ and the critical force F_krit_ were calculated from the experimental part (3102 N) to determine the reliability of the method. The difference between the forces obtained by the experimental and numerical methods was 7%. This difference was assessed as acceptable.

**Figure 8:**
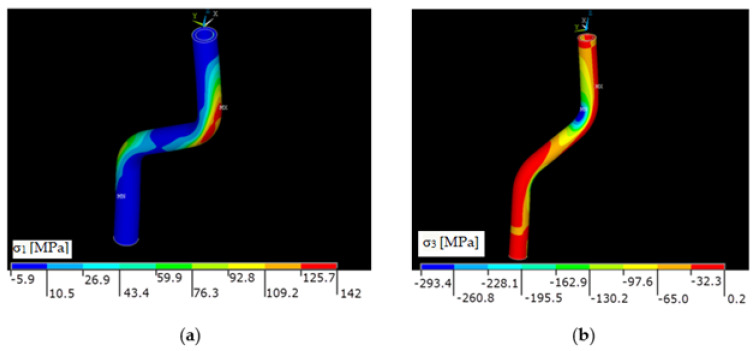
(**a**) Distribution of the first principal stress σ_1_ on the composite part in (MPa), (**b**) DistribuTable 3. on the composite part in (MPa).

The stress distributions from the static analysis for the load force 3100 N are shown in Figure 8a,b.

The greatest stresses occurred in the areas of bends, both for the walls and for the composite part of the profile. The first principal stress expresses the greatest stresses, and the third principal stress shows the lowest stresses, i.e., the stress without shear elements (normal stress). In this way, places with significant tensile or compressive stress can be detected, which can affect local strength, stability, or fatigue. Equivalent stress can mask these areas. 

### 3.3. Microstructure

#### 3.3.1. Microstructure of Composite Profiles Using Optical Microscopy

Images of the microstructure of the observed CF/onyx material were taken using an Olympus GX51 optical microscope with Image-Pro Premium 9.2 software for metallographic analysis.

**Figure 9:**
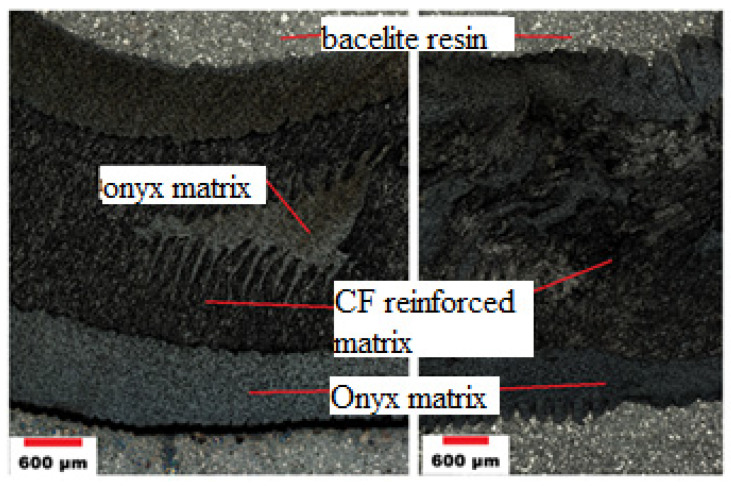
Optical microscopy; microstructure of CFRP/onyx produced by 3D printing. Undeformed sample (**left**), deformed sample (**right**).

Samples for metallographic analysis were taken from the composite profile perpendicular to the direction of the fibers. The sample for analysis is thus formed only by the cross-section of the fibers, as the printed profiles are reinforced with fibers in one direction. The carbon fibers coated with an onyx matrix are very strong in the longitudinal direction, especially in tension. The microstructure of the cut on the left is made of a straight part of the profile (Figure 9 (left)). On the right (Figure 9 (right)), is a picture of the microstructure of the sample taken from the bend of the profile, where a visible deformation occurred after the buckling test. Comparing the two figures, it is clear that the deformation caused waviness at the edges of the sample, where there is only a layer of onyx without reinforcing fibers. The middle layer of the onyx matrix is overlap by a deformed layer of carbon reinforcement.

#### 3.3.2. Microstructure of Composite Profiles Using SEM Analysis

For observation with SEM (Secondary Electron Microscope), Explorer 4 ThermoFisher Scientific with an accelerating voltage of 15 keV, the transverse surface of the samples had to be sprayed with Au-Pd conductive powder.

**Figure 10:**
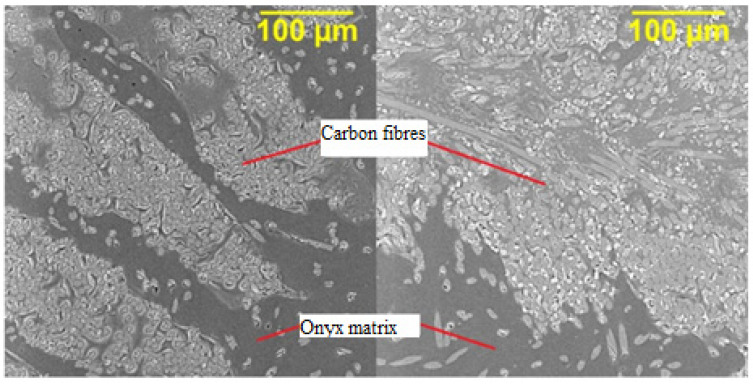
SEM analysis; microstructure of CFRP/onyx produced by 3D printing. Undeformed sample (**left**), deformed sample (**right**).

Figure 9 shows the microstructure of CFRP profiles made by 3D printing, where carbon fibers are printed into an onyx matrix. There is only a cross-section of the fibers in the microstructure, due to the one-way reinforcement along the length of the composite part. Figure 10 (left) shows the microstructure of a sample taken from an undeformed profile. Figure 10 (right) then shows the microstructure of the 3D printed profile after deformation. While in Figure 10 (left) there are places with missing reinforcement, after deformation, the surrounding composite material was compressed and accumulated in the originally unreinforced places (right).

## 4. Conclusions

This paper deals with the design and modeling of composite structures. The aim was to design, manufacture, and test an off-axis composite profile of circular cross-section. For 3D printed samples, FEM analysis revealed that the primary failure was not due to a loss of stability, but most likely to material failure. The stress limits for the limit state (LS) were calculated by static analysis, and the force required to achieve the LS was determined for these individual LS. The force value was calculated to be 2879 N. Under loading, several LS are expected to interact at once. Therefore, a procedure was proposed to determine the critical force from the simulation, the reliability of which differs only by 7% from the result of the experiment, 3102 N. In addition, it is on the safe side. The highest stresses were found in the same areas of the profiles where the material was significantly deformed during the experiment, in the place of hollow profiles. The failure was due to high stresses at the bends of the profiles and the interactions of several LS. The material failed, followed by a loss of stability due to the plastic joint.

The analyses created from FEM modeling can be used for approximate prediction of the critical force, especially for the buckling (and other mechanical properties) of composite profiles with respect to the properties of the fibers and resin used. The obtained results from the microstructure provide information on the quality of the final composite parts, especially porosity, insufficient fiber saturation, and corrugation. The connection between the occurrence of such defects in individual processing technologies makes it possible to set up the production process so that their occurrence is eliminated as much as possible. It is concluded from this article that the production of composite profiles using FDM method is geometrically accurate, production-repeatable, and these profiles are highly resistant to compressive deformations. Proposed investigations in the future include the development of experimental and numerical methods for the fatigue failure of an off-axis printed CFRP composite.

## Figures and Tables

**Table 1:** Mechanical properties of materials for CF/onyx profiles [43,44].

Composite Base (Matrix)	Test (ASTM)	Onyx
Tensile modulus (Gpa)	D638	1.4
Tensile Stress at Yield (MPa)	D638	40
Tensile Stress at Break (MPa)	D638	37
Tensile Strain at Break (%)	D638	58
Flexural Strength (MPa)	D790 ^1^	81
Flexural Modulus (GPa)	D790 ^1^	3.6
Heat Deflection Temp (°C)	D648 B	145
Izod Impact-notched (J/m)	D256-10 A	330
Density (g/cm^3^)	-	1.2
**Continuous Fiber**	**Test (ASTM)**	**Carbon**
Tensile Strength (MPa)	D3039	800
Tensile Modulus (GPa)	D3039	60
Tensile Strain at Break (%)	D3039	1.5
Flexural Strength (MPa)	D790 ^1^	540
Flexural Modulus (GPa)	D790 ^1^	51
Flexural Strain at Break (%)	D790 ^1^	1.2
Compressive Strength (MPa)	D6641	420
Compressive Modulus (MPa)	D6641	62
Compressive Strain at Break (%)	D6641	0.7
Heat Deflection Temp (°C)	D648 B	105
Izod Impact-notched (J/m)	D256-10 A	960
Density (g/cm^3^)	-	1.2

^1^ Measured by a method similar to ASTM D790. Composite Base—only parts do not break before end of flexural test.

**Table 2:** 3D printing parameters of CF/onyx profiles.

Dimensions	220 mm × 73 mm × 18 mm
Printing Temperature (onyx)	274 °C
Printing Temperature (CF)	252 °C
Layer height	0.125 mm
Number of layers	144
Fiber Fill Type	Isotropic Fiber
Fill Pattern	Triangular Fill
Fill Density	55%
Roof and Floor layers	2
Wall Layers	2
Print time	10 h 22 m
Plastic Volume	27.16 cm^3^
Fiber Volume	20.48 cm^3^
Final Part Mass	51.14 g
Plastic Angles	90° (not set one angle)
Fiber Angles	0°
Material cost	67.45 USD

**Table 3:** Results comparison of the maximum load force on the buckling test of hollow composite profiles.

n = 6	F_max_ (N)
Profile 1	3012
Profile 2	3248
Profile 3	3001
Profile 4	3095
Profile 5	3158
Profile 6	3096
Arithmetic mean	3102
Standard deviation	93
Coefficient of variation (%)	3

**Table 4:** Static analysis—material properties [44,49].

Onyx Wall	Young’s Modulus, E (MPa)	1400
	Poisson’s Ratio, μ	0.4
CF composite core	Matrix Young’s modulus, E_m_ (MPa)	1400
	Poisson’s ratio, μ_m_	0.4
	Fiber Young’s modulus, E_f_ (Mpa)	60,000
	Poisson’s ratio, μ_f_	0.33
	Fiber volumetric content, v_f_	0.77

**Table 5:** Static analysis—limit states.

Wall	1	Material Failure	Exceed of Strength Limit	36	3867
CF composite core	2	Tensile strength along the fibers	Fiber breakage	622	13,705
	3	Compressive strength along the fibers	Buckling of micro-fiber, Lo–Chim model	594	6309
	4	Tensile strength transverse to the fibers	Matrix failure in tension	36	2603
	5	Compressive strength transverse to the fibers	Matrix failure in shear	36	2168

## Data Availability

Not applicable.
